# Technical Note: Neural Network Architectures for Self-Supervised Body Part Regression Models with Automated Localized Segmentation Application

**DOI:** 10.1007/s10278-024-01319-z

**Published:** 2024-11-13

**Authors:** Michael Fei, Alan B. McMillan

**Affiliations:** 1 Creighton University School of Medicine, Phoenix, AZ USA; 2 University of Wisconsin-Madison, Madison, WI USA

**Keywords:** Artificial intelligence, Self-supervised learning, Segmentation, Deep learning

## Abstract

The advancement of medical image deep learning necessitates tools that can accurately identify body regions from whole-body scans to serve as an essential pre-processing step for downstream tasks. Typically, these deep learning models rely on labeled data and supervised learning, which is labor-intensive. However, the emergence of self-supervised learning is revolutionizing the field by eliminating the need for labels. The purpose of this study was to compare neural network architectures of self-supervised models that produced a body part regression (BPR) slice score to aid in the development of anatomically localized segmentation models. VGG, ResNet, DenseNet, ConvNext, and EfficientNet BPR models were implemented in the MONAI/Pytorch framework. Landmark organs were correlated to slice scores and mean absolute error (*MAE*) was calculated from the predicted slice and the actual slice of various organ landmarks. Four localized DynUNet segmentation models (thorax, upper abdomen, lower abdomen, and pelvis) were developed using the BPR slice scores. Dice similarity coefficient (*DSC*) was compared between the localized and baseline segmentation models. The best performing BPR model was the EfficientNet architecture with an overall 3.18 *MAE*, compared to the VGG baseline model with a *MAE* of 6.29. The localized segmentation model significantly outperformed the baseline in 16 out of 20 organs with a *DSC* of 0.88. Enhanced neural networks like EfficientNet have a large performance increase in localizing anatomical structures in a CT compared in BPR task. Utilizing BPR slice score is shown to be effective in anatomically localized segmentation tasks with improved performance.

## Background

Deep learning holds immense potential for processing extensive whole-body datasets, which are increasingly prevalent through the use of CT, PET/CT, MRI, and PET/MRI. Yet, with the sheer volume of data, a challenge lies in the specificity of many critical applications that require focus on particular body parts or organs. Consequently, the ability to rapidly and accurately determine the anatomical location through body part identification within these vast datasets is highly valuable. Such capability could enable rapid quality assurance checks to verify the correct body area is present, before resorting to computationally intensive procedures. Furthermore, such tools could enable the efficient routing of specific data subregions to the appropriate downstream algorithms, whether for detailed segmentation, acute trauma detection, or comprehensive body composition analyses. While segmentation algorithms can provide such anatomical information, there could exist extensive computing requirements which may not be feasible to do at scale. Therefore, the development of rapid and reduced resource approaches is likely to be useful.

A potential upside of body part identification can be realized with greatly reduced effort if no manual labeling is required using a self-supervised deep learning approach, whereas segmentation generally requires supervised ground truth labels. Classically, training a supervised deep learning neural network requires matched input data and manually obtained labels [1]. As computing hardware has rapidly advanced over the past decade larger and stronger models have been created to solve various tasks [2]; however, the biggest bottleneck is acquiring labeled data since it requires an enormous amount of manual labor [3]. Self-supervised deep learning is a powerful technique that does not require any labels [3]. Usually, self-supervised deep learning consists of a task that is an inherent part of the data. One example is rotating a training image and predicting the correct orientation [4]. Another common example is pre-training, realized by cropping out patches in training images and predicting what was cropped out [5]. Although these are relatively trivial tasks, certain characteristics of the data can be understood, and various features of the dataset are learned. This could lead to better transfer learning for a supervised task or any other downstream task with less labeled data required.

Yan et al. proposed a self-supervised convolutional neural network (CNN) model for body part identification based upon a VGG network [6]. The model was trained to predict the relative position of the Z slices in a volume. The model outputs a continuous score for each slice representative of the relative position in the body, the lower the slice score, the more inferior the slice within the body, and the higher the slice score, the more superior the slice within the body. Of note, the VGG model architecture was first proposed by Simonyan et al. in 2014 [7]. The VGG architecture presents as a potential limitation of this study as more powerful neural network architectures have been created which have been shown to yield better results in various biomedical applications like ResNet [8], DenseNet [9], ConvNeXt [10], and EfficientNet [11]. This study incorporated additional techniques which yielded stronger performance, like random augmentations, result post-processing, and a more detailed evaluation. These neural networks are not only deeper but also include various architecture improvements like residual layers [8], dense layers [9], and fine-tuned network architectures [11]. Finally, this work extends on the application of the BPR pipelining into a localized whole-body segmentation task. Other works have tried localizing CTs either through smaller segmentation models or bounding boxes [12, 13]. However, these solutions are limited by their generalizability and use more resources than a regressor score. We propose the BPR as an elegant solution for localization that can be generalized and scale to an infinite number of anatomical locations. The objective of this work is to study these enhanced neural network architectures for the self-supervised body part regression task and develop a novel pipeline to anatomically localized segmentation models using the BPR slice scores.

## Methods

### Dataset

The Pediatric-CT-SEG dataset from The Cancer Imaging Archive was used [14]. This study used an open-source dataset, thus ethical approval is not required. There was a total of 359 CTs with expert labels of various organ contours. Patient age ranges were 5 days to 16 years old with 180 males and 179 females. The images were acquired when clinically indicated, meaning the data is a mixture of normal and pathological cases. The model was split into 288/32/39 train/evaluation/test, respectively.

### BPR Neural Network Architecture

**Fig. 1:**
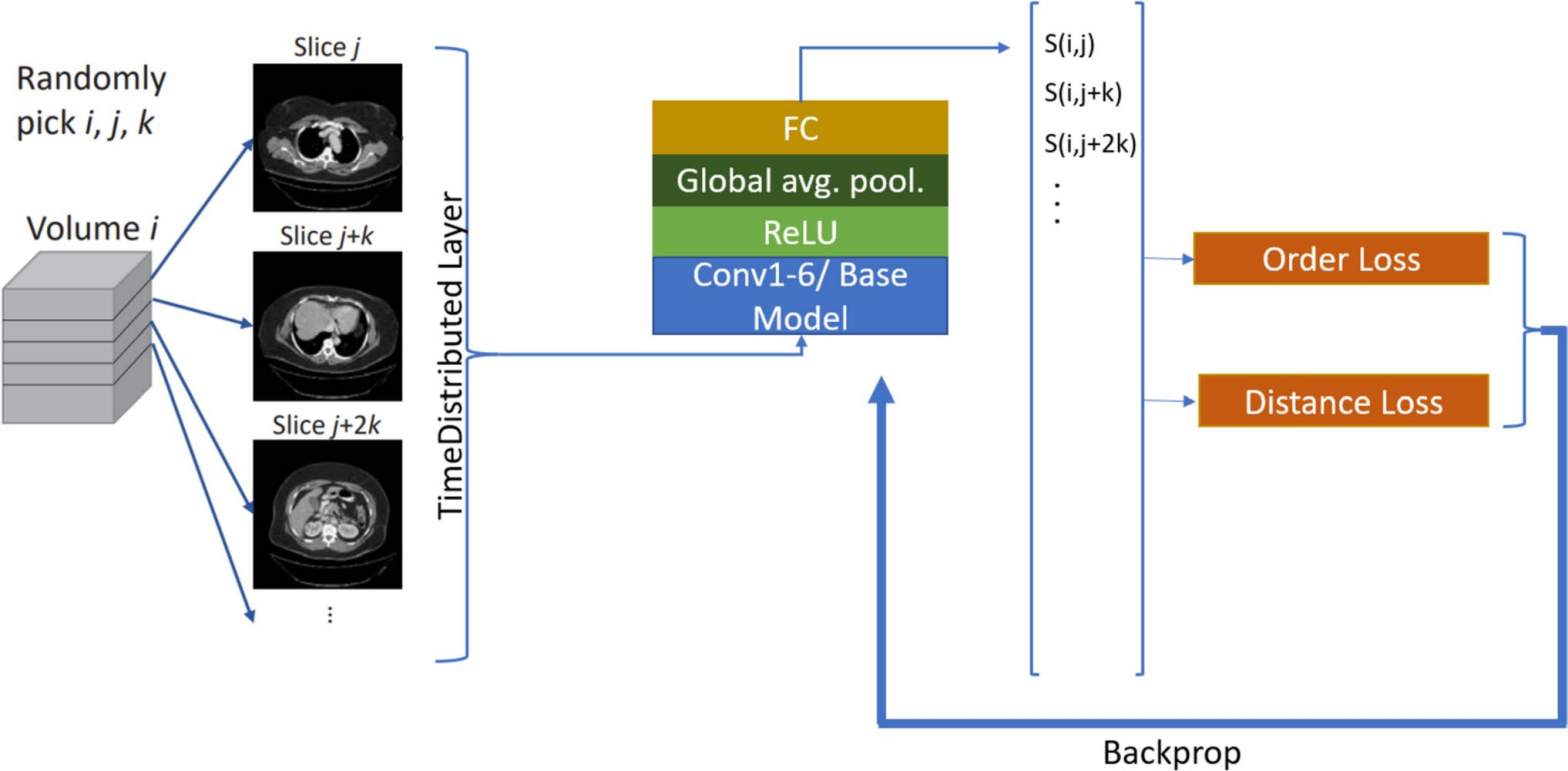
Pipeline of how slices are selected, and how model was trained. Conv1-6 is the VGG model from Yan et al. Other base models were ResNet50, DenseNet169, EfficientNet_v2_s, and ConvNeXt_small

All models were created using the MONAI [15] and Pytorch [16] frameworks. A total of 5 different network structures were compared. For all models, the pretrained weights from Torchvision [17] were loaded in. The VGG16 network as originally utilized by Yan et al. [6] was re-created to serve as the baseline. This model consisted of an additional convolutional layer with 512 1 × 1 filters, and stride 1 was added after the *conv5* layer which was followed by a ReLU layer and a global average pooling layer. Finally, a fully connected layer was created to give the final slice score. For the other 4 models, ResNet50, DenseNet169, EfficientNet_v2_s, and ConvNeXt_small, a similar procedure is used to adapt these network structures, as shown in Fig. 1. A ReLU layer was wrapped around the last layer of the based model, followed by a fully connected layer. All models were wrapped in a Pytorch time distributed layer which allows the models to be stacked [16]. Each model’s input was multiple slices, and the model outputs slice scores for each of those slices. The loss function considers an axial reformat of the slices within a single patient’s image volume.

### BPR Loss Function

The loss function consists of two parts, the order loss, and the distance loss. The order loss ensures that the score increases along the volume, while the distance loss ensures that the score increases linearly.

The order loss is:

$${L}_{order}=-\sum_{i=1}^{g}\sum_{j=1}^{m-1}\text{log}h(S\left(i,j+1\right)-S(i,j))$$

where *g* represents the total number of volumes in the batch; *m* is the number of selected axial slices in each image volume (CT, MRI, etc.); *h* is a sigmoid activation function; and* S*(*i, j*) is the slice score in the *i*^th^ volume respective to the *j*^th^ slice. This loss function constrains the model to produce higher slice scores for slices more superior within the volume as a large difference between the slice score of the slice above and the slice score of the slice below produces a low loss.

The distance loss is:

$${L}_{dist}= -\sum_{i=1}^{g}\sum_{j=1}^{m-2}f({\Delta }_{i,j+2}-{\Delta }_{i,j+1})$$

$${\Delta }_{i,j}= S\left(i,j\right)-S(i,j-1)$$

where *g* represents the total number of volumes in the batch; *m* is the number of selected slices in each volume; *f* is a smooth L1 loss; and *S*(*i, j*) is the slice score in the *i*^th^ volume on the *j*^th^ slice. This function constrains the model to produce slice scores that are evenly spaced. When the difference in the spacing between the two sets of slices is small, the loss is also small.

The final loss is a combination of the order loss and distance loss:

$$L= {L}_{order}+{L}_{dist}$$

These two loss functions ensure that the slice scores that are most inferior are smaller and linearly increase as the anatomical location moves superiorly.

### BPR Model Training

First, the data went through a series of random transformations or augmentation, which were not applied in the original study. These augmentations artificially created new sample images that the model has never seen which should increase its ability to generalize to novel images and the transformations also reduce overfitting. The augmentations applied to the images were randomly zoomed between 80 and 120% of their original size, randomly flipped across their *x* and *y* axis, randomly rotated 0.1 radians, randomly scaled in intensity between 80 and 120%, randomly shifted in intensity with between 0 and 0.08 and randomly applied Gaussian noise with a mean of 0.0 and a standard deviation of 0.04. A series of 8 random equidistance slices were chosen from a random image volume, *i*. First, a value, *k* was randomly chosen between 1 through 4 representing the spacing between the set of slices. A random number, *j*, is chosen between 1 through *t*-8*k* with *t* being the total number of slices in that image volume. This ensures that a choice is within the range of that image volume. The set of slices will be *j* + *k*(*x* − 1) with *x* being the *x*^th^ slice in the set of the 8 slices which can be visualized in Fig. 1. This randomly chosen subset of samples was chosen during the start of each epoch. The random slice sampling allowed the model to generalize further beyond its immediate nearby slices and also added more variability in training data accounting for various slice thicknesses. The image pixel values were normalized between 0 and 1 and resized to 128 × 128. The model was trained for 400 epochs with a batch size of 16 and the RMSprop optimizer with the learning rate of 1e − 4. The training was done on an NVIDIA RTX 3080 on a workstation with 32 GB of RAM.

### BPR Evaluation

Based on the labels from the Pediatric-CT-SEG dataset, the slice of the first occurrence of an organ and the last slice of the organ were extracted for the femoral head, rectum, bladder, large intestine, small intestine, kidney, liver, duodenum, gallbladder, pancreas, spleen, stomach, adrenal glands, lung, esophagus, and heart. This yielded a total of 32 organ landmarks. Due to the model and the loss functions, the model has no constraints on the range of slice scores it can produce. To normalize the slice score outputs, numbers were scaled using the following equation:

$$Scor{e}_{new}=\frac{(Scor{e}_{old}-MinValue)100}{MaxValue-MinValue}$$

where *MinValue* and *MaxValue* represent the pre-normalized mean slices score for the lowest landmark, the start of the femoral end, and the highest landmark, the end of the esophagus.

Next, the outputs were also passed through a Gaussian filter to smooth out the curve with a standard deviation of 10. Using the training dataset, the post-processed slice scores and the slices of each organ landmark, the average post-processed slice scores were found for each organ landmark. These reference scores were then used on the testing dataset to predict the slice of each organ landmark. Since the number of slices for each volume varies, the slices were all scaled so that the largest value was 100. The *MAE* was calculated between the true slice and the adjusted predicted slice. To assess statistical significance, a pairwise Wilcoxon test was performed between all models, and significance was corrected for using the Bonferroni correction. There was a total of 10 comparisons, so the Bonferroni-corrected *p* value was 0.005.

Additionally, a subset of 61 subjects released by TotalSegmentator [12] were used to externally validate the BPR model. This dataset consists of pathological and non-pathological adult CTs that the model has never seen. Using the generated landmark values from the EfficientNet model, the *MAE* between the true and the adjusted predicted slice were calculated. Some organ landmarks were excluded due to the lack of labels or different label definitions.

### Anatomically Localized Segmentation Experiment

Using the same train/evaluation/test split, anatomically localized segmentation models were developed to utilize the regression scores. The whole body CT was split into four sections, thorax (heart, esophagus, left lung, and right lung), upper abdomen (liver, stomach, pancreas, spleen, left adrenal, right adrenal, gall bladder, left kidney, and right kidney), lower abdomen (duodenum, small intestine, and large intestine), and pelvis (bladder, rectum, left femoral head, and right femoral head). Using the EfficientNet BPR model, a BPR slice score was generated for each slice of all CTs. At each organ landmark, the BPR slice score at that slice was average across the training dataset. This correlated BPR slice scores to anatomical locations. For each of the four sections, the CTs were then cropped 1.5 standard deviations below and above the BPR slice scores of the highest and lowest landmark organs, respectively. For example, the thorax section is bounded by the top of the heart and the bottom of the lungs, thus the CT was cropped 1.5 standard deviations above the top of the heart and 1.5 deviations below the bottom of the lung. The 1.5 standard deviations allowed for the majority of regions interested to be captured even with anatomical variations. Image pixel values were normalized between zero and one. The Monai foreground crop was used in the training process and pixel spacing was readjusted to 1.5 × 1.5 × 2. DynUNet, based around nnU-net [18], with a 48 × 48 × 48 input size was developed and trained with 48 × 48 × 48 randomly cropped patches. The models used a dice cross entropy lost function and trained with the AdamW optimizer with a learning rate of 1e-4 and a weight decay of 1e-5 over 500 epochs. Dice similarity coefficient (*DSC*) of the whole CT was used to assess the performance with cropped out regions as background, along with 95% confidence intervals. Four baseline models were trained with the same organs where the CTs were not cropped using BPR and trained on the whole body. *DSC*s were compared for each segmentation model, as well as with previous literature’s best segmentation values. A Wilcoxon test was performed between the localized and whole-body segmentation on each of the segmented landmark. The significance was corrected for using the Bonferroni correction. There were a total of 20 comparisons, so the Bonferroni-corrected *p*-value was 0.0025, as there were 20 comparisons. Finally, a Wilcoxon test was performed comparing the average scores of the localized and whole-body segmentations with a *p*-value of 0.05.

## Results

**Fig. 2:**
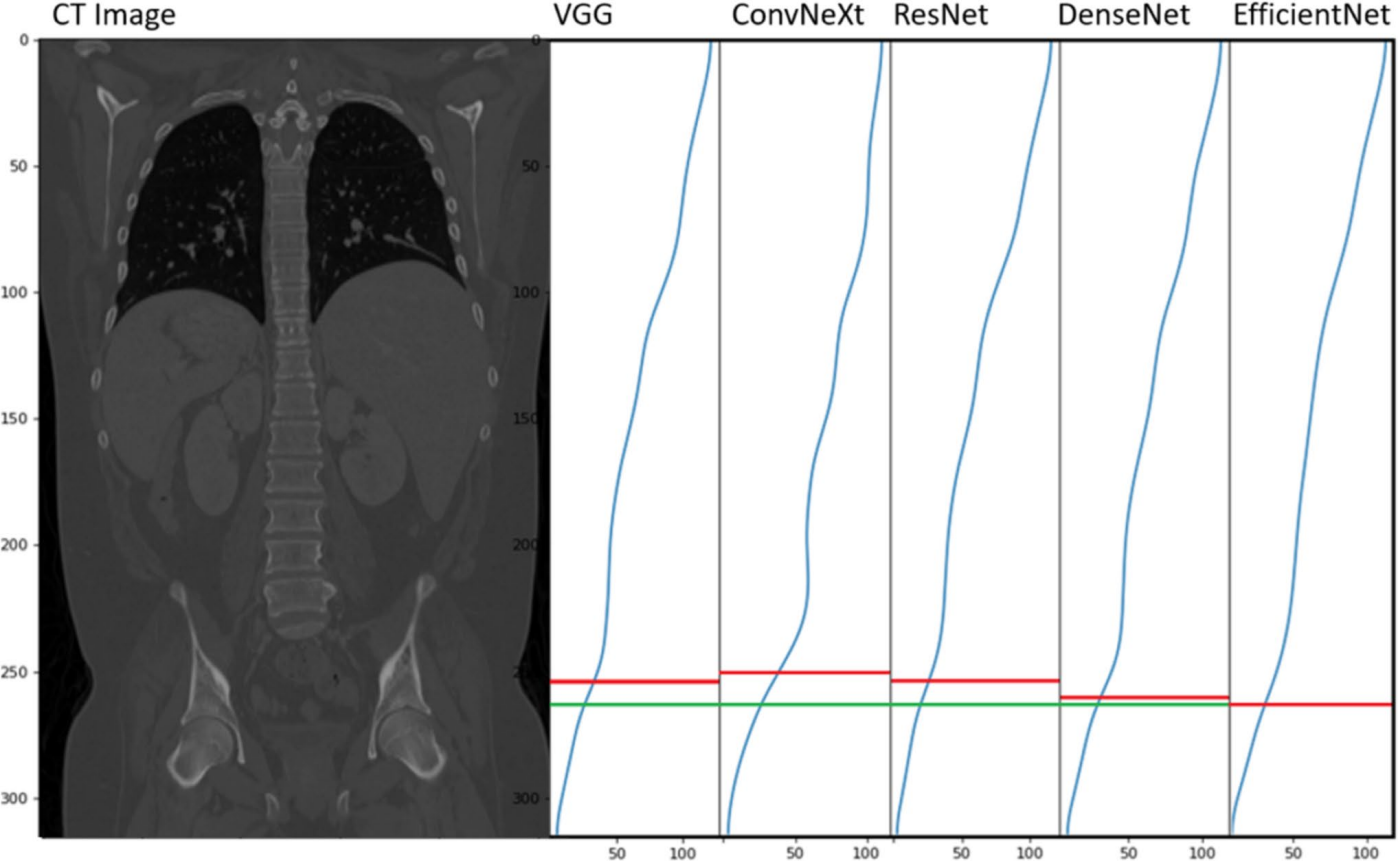
Sample output of each model with the example of the end of the femoral head landmark. The green denotes the true ending slice, and the red denotes the model predicted slice. The *X* axis on each graph is the BPR score

**Fig. 3:**
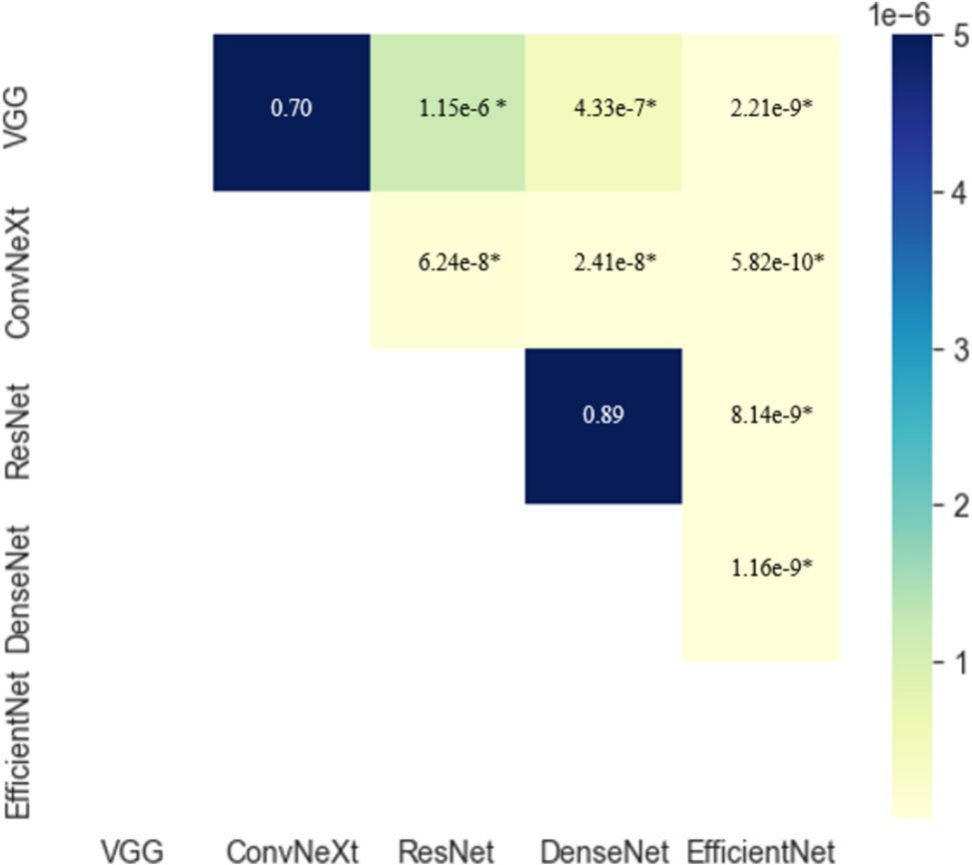
Pairwise results of Wilcoxon test of the *MAE* of the test dataset with respective *p*-values. * denotes *p* < 0.005

For each of the 5 models, the average *MAE* for all landmark organs is shown in Table 1. The overall *MAE* for the test dataset of VGG, ConvNeXt, ResNet, DenseNet, and EfficientNet are 6.29, 6.40, 4.44, 4.41, and 3.18, respectively, as shown in Table 1. EfficientNet performed the best overall with the lowest *MAE* on both the evaluation and test datasets. It also performed the best on 30 out of the 32 landmark organs for the evaluation dataset and 28 out of the 32 landmark organs on the test dataset. On the 4 landmarks that other models outperformed on for the test dataset, there were marginal differences in the *MAE*. EfficientNet had the best performance on the spleen end, femoral end, stomach end, and heart start with *MAE* 1.36, 1.39, 1.50, and 1.80, respectively. It had the worst performance on the esophagus end, lung end, and bladder end with *MAE* of 8.10, 6.01, and 5.29, respectively. Although all models generated a slice score curve that increase relativity monotonically, the EfficientNet creates a curve that generalizes best to the anatomical landmarks, as shown with the example in Fig. 2 which displays sample output slice scores for each model with an example of the label anatomical location and the predicted anatomical location. Figure 3 shows EfficientNet statistically performed significantly better than all other models. DenseNet and ResNet showed similar performance which outperformed VGG and ConvNeXt. ConvNeXt showed similar performance to the baseline VGG model. Finally, the TotalSegmentator data set had an overall *MAE* of 4.67. It showed similar performance with adrenal end, esophagus start, stomach end, and heart start with *MAE* of 2.57, 2.68, 2.71, and 2.77, respectively. The worst performing sections were lung end, esophagus end, and duodenum start with 7.42, 7.00, and 6.63.

**Table 1:** *MAE* of each organ landmark for each model on the evaluation and test dataset

Models		VGG		ConvNeXt		ResNet		DenseNet		EfficientNet		
Landmarks		Val	Test	Val	Test	Val	Test	Val	Test	Val	Test	TotalSeg
Femoral head	Start	4.35	3.60	3.75	2.72	3.69	3.16	3.71	3.04	**2.32**	**1.91**	
	End	5.09	5.24	2.47	1.98	3.80	2.70	3.57	2.72	**1.71**	**1.39**	
Rectum	Start	3.22	3.87	2.52	**2.60**	2.94	3.49	3.16	3.62	**2.08**	2.71	
	End	6.40	5.97	3.69	3.38	5.03	4.01	4.86	3.69	**3.40**	**2.54**	
Bladder	Start	4.45	4.96	2.96	2.82	3.69	3.94	3.72	3.71	**1.88**	**2.25**	
	End	7.49	8.56	5.14	5.44	5.67	6.26	5.77	6.08	**4.93**	**5.29**	
Large intestine	Start	5.47	5.60	2.85	3.11	3.84	3.87	4.02	3.87	**2.75**	**2.79**	5.20
	End	8.21	8.47	8.86	10.11	6.55	5.88	6.54	6.08	**4.78**	**4.56**	4.73
Small intestine	Start	7.90	8.25	4.97	3.81	5.93	4.83	6.19	4.60	**4.79**	**3.61**	3.43
	End	8.16	5.60	9.26	8.52	6.15	5.07	6.07	5.18	**3.86**	**3.45**	5.40
Kidney	Start	6.10	6.28	6.85	7.75	5.97	6.06	5.93	5.71	**4.19**	**4.08**	4.15
	End	7.90	6.52	9.31	7.85	5.49	3.81	5.57	4.27	**3.11**	**2.24**	3.90
Liver	Start	6.80	7.02	7.32	8.17	6.74	6.48	6.59	5.99	**4.99**	**4.34**	5.03
	End	4.32	3.85	4.11	4.04	2.32	2.49	2.63	2.18	**2.15**	**1.85**	3.50
Duodenum	Start	6.48	6.46	6.85	8.00	6.00	6.05	5.85	5.91	**4.12**	**4.29**	6.63
	End	7.48	6.21	8.98	7.71	5.69	3.86	5.81	4.37	**2.57**	**2.28**	4.90
Gall bladder	Start	7.24	6.61	7.17	7.66	5.73	5.98	5.75	5.99	**4.57**	**4.31**	4.80
	End	7.21	6.77	8.70	8.09	5.48	3.91	5.74	4.60	**2.95**	**2.47**	4.72
Pancreas	Start	7.36	6.27	7.94	6.36	5.77	5.05	5.89	4.85	**3.72**	**3.34**	6.29
	End	7.58	6.38	8.60	8.26	5.24	3.85	4.95	4.32	**2.62**	**2.17**	3.90
Spleen	Start	8.96	4.49	7.84	8.43	5.90	7.00	5.97	6.71	**3.59**	**4.81**	3.96
	End	4.81	6.08	5.74	4.67	2.78	2.23	2.31	1.78	**1.79**	**1.36**	3.26
Stomach	Start	9.44	8.69	7.61	8.06	6.24	6.23	6.07	6.03	**5.16**	**4.77**	5.91
	End	4.98	4.82	6.43	5.38	2.97	2.28	2.65	2.03	**1.72**	**1.50**	2.71
Adrenal	Start	8.65	8.21	10.03	8.70	6.61	5.19	6.93	5.50	**4.07**	**3.25**	6.19
	End	7.09	5.89	8.64	8.00	5.09	3.71	4.80	4.07	**2.38**	**2.18**	2.57
Lung	Start	6.81	6.11	8.58	8.16	4.45	3.40	4.18	3.53	**1.73**	**1.85**	4.70
	End	7.37	7.70	8.11	7.20	**5.27**	5.58	**5.11**	5.33	5.36	6.01	7.42
Esophagus	Start	6.35	5.23	7.92	7.29	3.73	3.52	3.42	3.37	**2.08**	**2.05**	2.68
	End	9.68	9.43	10.53	10.02	7.64	7.69	**7.39**	**7.36**	7.74	8.12	7.00
Heart	Start	5.44	4.60	6.31	6.41	2.90	2.59	2.47	2.45	**1.86**	**1.80**	2.77
	End	4.90	4.04	6.03	4.02	3.15	**1.95**	3.49	2.18	**3.00**	2.28	5.84
Overall		6.68	6.29	6.75	6.40	4.95	4.44	4.91	4.41	**3.37**	**3.18**	4.67

The bold numbers represent the lowest *MAE* for each model

**Fig. 4:**
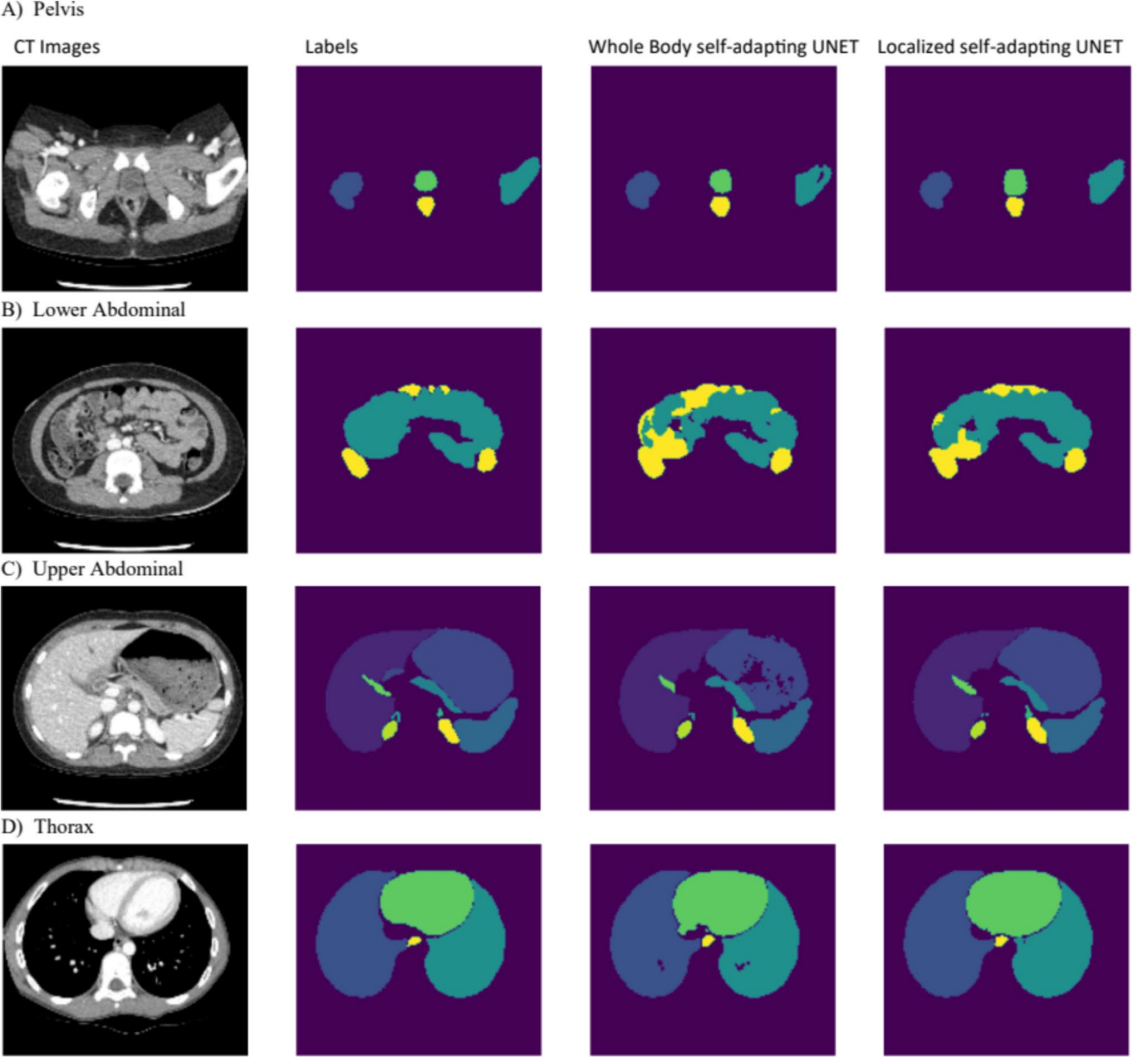
Sample slices from each model comparing the CT images, labels, whole body self-adapting UNET output and localized self-adapting UNET output. **A** shows the left and right femoral head, bladder and rectum. **B** shows the small and large intestines. **C** shows the left and right adrenal glands, left and right kidneys, liver, stomach, pancreas, and gallbladder. **D** shows the left and right lung, heart, and esophagus

Localized models show a significant increase in model performance on 14 of the 20 segmented organs compared to the baseline whole body segmentation model. *DSC* is shown in Table 2. When comparing the overall dice scores of localized and the whole body, the localized model showed a significant increase in dice with an overall dice of 0.85 compared to a 0.81 and a *p*-value of 1.9e − 6. Sample segmentation results are shown in Fig. 4. The reference by Adamson et al. did not segment the adrenal glands, so when comparing *DSC* using the same organ subset the localized model outperformed the reference implementation with 0.88 compared to 0.84 *DSC* [19].

**Table 2:** Average dice score for each organ with VNET model from literature, model trained with whole-body CT, and the model trained with localized CT

Region	Anatomical structure	Reference (VNET)	Whole body self-adapting UNET	Localized self-adapting UNET
Thorax	Heart	0.94	0.93 [0.91, 0.94]	0.94 [0.93, 0.95]
	Esophagus	0.68	0.68 [0.64, 0.71]	**0.75** [0.72, 0.78]
	Lung right	0.97	0.97 [0.96, 0.97]	0.97 [0.97, 0.98]
	Lung left	0.97	0.95 [0.93, 0.97]	0.96 [0.94, 0.98]
Upper abdomen	Liver	0.96	0.97 [0.96, 0.97]	**0.97** [0.97, 0.98]
	Stomach	0.89	0.86 [0.81, 0.90]	**0.90** [0.87, 0.93]
	Pancreas	0.73	0.71 [0.68, 0.76]	**0.76** [0.71, 0.80]
	Spleen	0.95	0.93 [0.92, 0.95]	**0.95** [0.94, 0.96]
	Adrenal left		0.36 [0.29, 0.43]	**0.54** [0.48, 0.61]
	Adrenal right		0.53 [0.48, 0.58]	**0.60** [0.56, 0.65]
	Gall bladder	0.81	0.86 [0.82, 0.90]	**0.88** [0.85, 0.91]
	Kidney left	0.96	0.94 [0.91, 0.96]	**0.96** [0.94, 0.96]
	Kidney right	0.95	0.96 [0.95, 0.97]	0.96 [0.95, 0.97]
Lower abdomen	Duodenum	0.51	0.62 [0.57, 0.67]	0.64 [0.58, 0.69]
	Small intestine	0.75	0.80 [0.77, 0.83]	**0.83** [0.80, 0.85]
	Large intestine	0.73	0.80 [0.77, 0.83]	0.81 [0.78, 0.84]
Pelvis	Bladder	0.85	0.80 [0.71, 0.89]	**0.85** [0.77, 0.93]
	Rectum	0.73	0.72 [0.66, 0.79]	**0.80** [0.74, 0.85]
	Femoral head left	0.91	0.88 [0.84, 0.92]	**0.93** [0.90, 0.96]
	Femoral head right	0.92	0.87 [0.84, 0.89]	**0.93** [0.92, 0.95]
Overall*		0.84	0.85 (0.81)	**0.88 (0.85)**

Averages do not include the left and right adrenal. Values in parenthesis include the left and right adrenal. The bold values are where there is a statistically significant increase in dice score between the localized and the whole-body segmentation model. The values in brackets are the 95% confidence intervals

## Discussion

The integration of more modern deep learning architectures yielded better performance for the body part regression model with almost all tested models performing better than the baseline VGG architecture. Furthermore, a locally trained segmentation model yielded superior performance compared to one trained on the whole body simultaneously. The VGG architecture had 6.29 *MAE* on the test dataset, which is significantly larger than the *MAE* of the other models, except ConvNeXt, which had an *MAE* of 6.40. The best performing was EfficientNet with an *MAE* of 3.18, a 50.55% decrease from the VGG *MAE*. Since the number of slices was rescaled to 100, an overall 3.18 *MAE* suggests that the model on average is able to localize the landmark organ to 3.18% of the original CT. Depending on the organ landmarks, it on average can localize between 1.36% and 8.01% of the original CT. The BPR model utilized herein was able to generalize to an additional, unseen dataset (TotalSegmentator). The model was able to localize to within 4.67% of the CT scan on average. The purpose of this work was to demonstrate the potential of improvements to BPR that improve total body segmentation by utilizing more efficient and better performing localized models. Indeed, a BPR model could be readily trained using the TotalSegmentator dataset.

The original Yan et al. study evaluated their performance by reducing the task to a classification problem of chest, abdominal, and pelvis. However, this is generally an easier problem and has limited value in understanding the practical applications of the resulting slice scores. Our results suggest that EfficientNet is able to accurately localize both the start and end of different anatomical landmarks. Some of the worst performing landmarks were the esophagus end, large intestine end, and lung end. For the organ landmarks exiting more superiorly in the body, like the esophagus end and lung end, this could be because the model is trained on fewer data samples due to the random slice sampler having a lower probability of hitting the more superior slices. Running a Monte Carlo simulation with the used slice selection parameters showed 10% of the most superior slices were selected, and 43% less than the average number of times a slice was selected. Datasets with increased and more uniform coverage would likely improve this potential issue. Thus, the biggest contributor is likely due to variability in what is captured by the CT, and the end of some organ structures might be where the anatomical coverage of the CT ends rather than the true end of the organ landmark. Finally, for other structures like the large intestine end or bladder end, it could suggest high inter-patient variability on those anatomical landmarks, especially in a pediatric population where there is a large range in physiology depending on gender and age. Overall, with the increased ability to automatically localize specific body regions, it offers high potential value to whole-body processing pipelines. Additional information can be provided to improve pre-processing, perform curation, mitigate inherent class imbalance issues for small organs, perform out-of-distribution detection, and/or localize segmentation models. Whole-body regression could be extremely practical in smaller lightweight models as the dataset can be significantly reduced, lowering the features and mathematical relationship the model needs to learn. This theoretically allows the tradeoff to be made between runtime and hardware requirements without losses in performance.

Deep learning organ segmentation models have become larger allowing for segmentation to be performed on over 100 classes in a whole-body CT [12]. One problem with these models is that the size and capability may be limited by hardware resources since each *N* class outputs an L × H × W mask. To solve this, whole body segmentation models are often broken up into various smaller models [12]. The segmentation application with the BPR shown in this paper provides an elegant solution in clustering organ landmarks into models that minimizes output volume size while maximizing the number of output classes. The localized model approach gives strong performance with smaller memory requirements. The proposed model had a last SoftMax output 56 times smaller than the reference VNET. Segmentation models usually take in a patch size that is a small subset of the larger image volume, rather than the whole volume. Because of this, the model does not have information on where the patches are anatomically. Thus, a common cause of incorrect mask from deep learning models in whole body segmentation task are labels of organs in anatomically impossible areas [20]. The BPR solves this problem by constraining the model to only areas where the region of interest is expected. This additionally reduces runtime as the sliding window on the model’s input does not have to process the entire scan but only in the areas where region of interest is expected. Additionally, we hypothesize that this more focused training in the region of interest allows the model to learn more details within an area, shown by significant improvement in smaller and more complex landmarks like the adrenal glands and small intestines. This is also supported by improved dice score when incorporating BPR for a local segmentation model.

Deep learning-based localization methods have been explored in various studies. One approach employs bounding boxes to identify organs of interest [13]. However, bounding boxes pose scalability challenges, particularly when tasks require more than 100 segmentation outputs. When new regions of interest (*ROI*s) are introduced, entirely new models must be trained, limiting flexibility. Moreover, in many cases, segmentation masks are preferred over bounding boxes because they provide additional training benefits. For instance, in detection and medical grading tasks, segmentation masks can be used as an extra input channel, offering the model more contextual information about the *ROI*. In contrast, the BPR solution offers an elegant method to localize all body part regions in a whole-body CT scan in a single pass and can adapt to future *ROI*s without necessitating new models. Another approach to localization is to apply a rough segmentation model before running a final segmentation algorithm, as seen with TotalSegmentator [12]. However, BPR significantly reduces resource requirements, as rough segmentation models still need to generate output masks for each organ at the size of the imaging volume. In comparison, the BPR model generates a simple list of regression scores, making it far more efficient.

One limitation of the study is that only one dataset was used to train and test the model. This was specifically a pediatric dataset, so there could be varying results with adult population data where there are larger anatomical differences within. Additionally, as mentioned, the random slice sampler under sampled the superior slices. A less biased slice sampler could be developed to achieve a distribution where all slices pass through the model an equal number of times. Furthermore, an additional model could be developed for coronal and sagittal slices, although applications could be limited due to the smaller range.

## Conclusion

This study showed that modern neural networks do indeed outperform the VGG architecture with all the architectures tested having a lower *MSE* than the *MSE* of the VGG architecture. The best performing model was the EfficientNet being able to localize on average an organ landmark within 4% of the CT. Using the BPR score, the proposed segmentation model allows for scalable segmentation models to be created by focusing on local regions using an automated, self-supervised approach. Overall, this gives promising results that give the possibility to many other downstream tasks.

## Data Availability

The models ran and links to the data can be found at https://github.com/mfei1225/BPR.
